# Epidemiology of Macrovascular and Microvascular Complications Among Patients With Diabetes Mellitus in Primary Care in Bahrain

**DOI:** 10.7759/cureus.99184

**Published:** 2025-12-14

**Authors:** Mahmood Alawainati, Nora AlGhareeb, Wafa Najem, Muneera AlGhareeb, Reem Alhouli, Alaa Alqallaf, Mariam Buhejji, Aysha Almulla, Sarah Obaid, Jumana AlRabiah, Lujain Hussain

**Affiliations:** 1 Medicine, Royal College of Surgeons in Ireland, Manama, BHR; 2 Family Medicine, Primary Healthcare Centers, Manama, BHR; 3 College of Medicine and Medical Science, Arabian Gulf University, Manama, BHR; 4 Medicine, Salmaniya Medical Complex, Manama, BHR; 5 Ophthalmology, Ministry of Health, Kuwait, KWT; 6 Medicine, Almaarefa University, Riyadh, SAU

**Keywords:** acute coronary syndrome, diabetes mellitus, hypertension, peripheral arterial disease, retinal diseases, stroke

## Abstract

Introduction

Although diabetes mellitus (DM) is a leading cause of cardiovascular complications, few studies have evaluated the epidemiology of these complications in primary care in Bahrain. Since data from primary care settings are particularly important, as most patients with diabetes are managed in this setting, this study aimed to determine the prevalence and predictors of diabetic complications among patients attending primary healthcare centers in Bahrain.

Methods

A cross-sectional study was conducted among adult patients with DM attending diabetic clinics in primary care centers across Bahrain. Demographic, clinical, and laboratory data, and diabetes complications were collected. Microvascular (retinopathy, nephropathy, neuropathy) and macrovascular (stroke, acute coronary syndrome, peripheral arterial disease) complications were assessed. Descriptive and inferential analyses were performed.

Results

A total of 585 patients were enrolled, with a median age of 61±14 years. Most patients were males (n=326, 55.7%), had type 2 DM (n=566, 96.8%), and had uncontrolled diabetes (n=349, 59.7%). Approximately half of the patients (51.6%) had at least one diabetes-related complication. Neuropathy (n=158, 27.0%) and retinopathy (n=89, 15.2%) were the most frequent microvascular complications, while peripheral vascular disease (n=64, 10.9%) and ischemic heart disease (n=61, 10.4%) were the leading macrovascular complications. Multivariable analysis revealed that higher total cholesterol (OR=1.326, p=0.031) and lower estimated glomerular filtration rate, eGFR (OR=1.009, p=0.025) predicted macrovascular complications. Diabetic foot ulcers increased macrovascular complications by 14-fold (p<0.001). Predictors of microvascular complications included type 2 DM (OR=16.277, p=0.011), longer DM duration (OR=1.156, p<0.001), higher triglycerides (OR=1.222, p=0.045), dyslipidemia (p=0.036), and foot ulcers (27-fold; p=0.002).

Conclusion

Diabetic complications were prevalent among diabetic patients in Bahrain, especially those with impaired renal function, dyslipidemia, anemia, and foot ulcers. Early identification and management of these comorbidities is crucial to prevent diabetes-related complications.

## Introduction

Diabetes mellitus (DM) is a chronic metabolic disorder that is increasing in prevalence worldwide. It is now considered a global pandemic. Epidemiological studies reported that DM affected almost 540 million adults globally in 2024 [1]. Due to cultural factors and genetic predisposition, the Gulf Cooperation Council (GCC) countries have some of the highest rates of DM in the world, along with a correspondingly high burden of its complications and sequelae [2].

DM is associated with high rates of morbidity and mortality. In general, the complications of DM are classified into two main categories: macrovascular and microvascular complications. This classification reflects the impact of diabetes on both large and small blood vessels, leading to a wide range of cardiovascular, renal, neurological, and ocular disorders [3]. Chronic hyperglycemia causes metabolic and structural changes in vascular tissues, resulting in endothelial dysfunction and increased vascular permeability [4]. Consequently, accelerated atherosclerosis leads to macrovascular complications, including ischemic heart disease, cerebrovascular attack, and peripheral vascular diseases, while capillary occlusion, leakage, and ischemia cause microvascular complications, i.e., retinopathy, nephropathy, and neuropathy [5]. Early detection and management of these complications is essential to reduce disease burden [3-5]. 

The literature showed variable prevalence of microvascular and macrovascular complications among patients with DM. Globally, a systematic review of 33 studies involving 13,283 participants from low- and middle-income countries revealed neuropathy prevalence of 16%, retinopathy prevalence of 12% and nephropathy prevalence of 15%. The study also found that ischemic heart disease affected 10%, peripheral arterial disease (6%), and stroke (2%) [6]. Another study of more than 11,000 patients with type 2 DM from 33 countries found that 19% had microvascular and 13.2% had macrovascular complications, with higher rates among patients who had higher glycosylated hemoglobin (HbA1c), smokers, and had previous macrovascular complications [7]. A retrospective cohort study of 135,199 patients with type 2 DM in the US found that older age, higher glycated hemoglobin (A1c), smoking, and hypertension were factors associated with macrovascular and microvascular complications of DM [8]. A cross-sectional study of 422 patients with type 2 DM in Tanzania found that 57.6% had microvascular complications, with diabetic retinopathy being the most common (21.1%). The study also showed that irregular physical activity, obesity, hypertension, longer disease duration, and inconsistent use of anti-diabetic medications were significantly associated with these complications [9].

Regionally, a cross-sectional study in Egypt, involving 506 patients, found that peripheral neuropathy affected 20% of patients, nephropathy affected almost one third of patients, retinopathy was observed in 35%, and peripheral arterial disease (PAD) in 32.6% of DM patients [10]. In Saudi Arabia, a national cross-sectional survey of 1240 diabetic patients found a 6.05% prevalence of micro and macrovascular complications, with myocardial infarction (3.5%), stroke (1.2%), and renal failure (1.9%) being the most common [11]. Another study of 1121 participants conducted in Saudi Arabia revealed that retinopathy (42.8%) and neuropathy (20.3%) were the most common microvascular complications, while coronary artery disease and peripheral arterial disease were the most common macrovascular complications (17.0% and 13.1%, respectively) [12]. According to these two studies, increasing age, smoking, hypertension, obesity, poor glycemic control, and low physical activity were significant factors associated with diabetes complications.

Although the prevalence and burden of DM have been increasing globally and in Bahrain, no previous studies have assessed the epidemiology of macrovascular and microvascular complications of DM [13]. In addition, data from primary care settings are particularly important, as most patients with diabetes are managed in this setting. Therefore, this study aimed to determine the prevalence and risk factors of diabetic complications among diabetic patients in primary care in Bahrain.

## Materials and methods

This cross-sectional study included patients attending the diabetic clinics at primary care centers in Bahrain between October and November 2025. Ethical approval was obtained from the Primary Healthcare Research Committee (PHCRC/TOR/0034/2025, dated 05/11/2025).

Patients aged 18 years and older with a labeled diagnosis of DM attending diabetic clinics in primary care centers in Bahrain were included. Individuals with emergency conditions, active cognitive impairment, psychiatric disorders, or language barriers were excluded from the study. Patients with type 1 and type 2 DM were both included.

The sample size was calculated based on the following variables: the maximum prevalence of DM complications based on literature (~58), a 95% confidence interval (CI), and a 5% margin of error. The sample size was calculated to be approximately 400 patients. To increase the power, a sample size of >500 was targeted. In Bahrain, there are 26 centers distributed over four governorates. All patients attending diabetic clinics at the time of data collection were enrolled. The period of data collection was selected randomly based on a draw.

Demographic data, including age, sex, and nationality, as well as risk factors such as dyslipidemia, hypertension, smoking, and alcohol consumption, and laboratory tests including A1c, fasting plasma glucose, lipid profile, and renal function tests, were collected. All information on microvascular and macrovascular complications of diabetes was obtained. DM was diagnosed according to the American Diabetes Association (ADA) criteria.

Microvascular complications included retinopathy, nephropathy, and neuropathy, while macrovascular complications included stroke, acute coronary syndrome, and peripheral arterial disease. Retinopathy was diagnosed based on retinal assessment done by a specialized optometrist, nephropathy was diagnosed based on the estimated glomerular filtration rate (eGFR<60 mL/min/1.73 m² for ≥3 months), and neuropathy was diagnosed based on monofilament (missing sensation in ≥1 sites) and clinical symptoms, while stroke and acute coronary syndrome were assessed by history. Patients were asked if they had a stroke or acute coronary syndrome in the past (self-reported stroke and acute coronary syndrome were verified using medical records).

The data were analyzed using the Statistical Package for the Social Sciences software version 28 (SPSS, IBM Corporation, Armonk, USA). Frequencies and percentages were used for categorical variables, while median with interquartile range (IQR) were used for continuous variables. The Mann-Whitney test was used to compare continuous variables, while the Chi-square test or Fisher's exact test was used to test the association between the categorical variables. A multivariable logistic regression model was developed, and the final step of the order regression method was reported. Variables associated with diabetic complications in the bivariate analysis at p<0.05 were entered into a multivariable logistic regression model. For all statistical analyses, statistical significance was set at p<0.05.

## Results

A total of 585 patients were enrolled with a median age of 61±14 years. Males (n=326, 55.7%) and Bahraini patients (n=499, 85.3%) constituted the majority of participants. Most patients had type 2 diabetes (n=566, 96.8%), dyslipidemia (n=455, 77.8%), and hypertension (n=388, 66.3%). Less than half of the patients had controlled diabetes (n=236, 40.3%), and the median age of diabetes duration was 11±13 years.

Regarding diabetes-related complications, more than half of the diabetic patients experienced one or more complications related to their condition (n=302, 51.6%). Specifically, neuropathy was the most common complication (n=158, 27.0%), followed by retinopathy (n=89, 15.2%) and nephropathy (n=84, 14.4%). Additionally, peripheral vascular disease was observed in 64 patients (10.9%), while ischemic heart disease and stroke were noted in 10.4% (n=61) and 5.8% (n=34) of patients, respectively. Table 1 presents the baseline characteristics of the patients.

**Table 1:** Demographic, clinical, and diabetes-related complications among the participants

Variable		n (%)
Sex	Male	326 (55.7)
	Female	259 (44.3)
Nationality	Bahraini	499 (85.3)
	Non-Bahraini	86 (14.7)
Smoking	Yes	78 (13.3
	No	459 (78.5)
	Ex-smoker	48 (8.2)
Alcohol	Yes	12 (2.1)
	No	566 (96.8)
	Ex-drinker	7 (1.2)
Type of diabetes	1	19 (3.2)
	2	566 (96.8)
Insulin	Yes	211 (36.1)
	No	374 (63.9)
Metformin	Yes	458 (78.3)
	No	127 (21.7)
Statins	Yes	468 (80)
	No	117 (20)
Dyslipidemia	Yes	455 (77.8)
	No	130 (22.2)
Hypertension	Yes	388 (66.3)
	No	197 (33.7)
Diabetic control	Yes	236 (40.3)
	No	349 (59.7)
Diabetic foot ulcers	Yes	20 (3.4)
	No	565 (96.6)
Diabetes-related complications	Any complication	302 (51.6)
	Neuropathy	158 (27.0)
	Retinopathy	89 (15.2)
	Nephropathy	84 (14.4)
	Peripheral vascular diseases	64 (10.9)
	Ischemic heart disease	61 (10.4)
	Stroke	34 (5.8)

As shown in Table 2, insulin use was significantly more prevalent among patients with macrovascular (p=0.023) and microvascular (p<0.001) complications. The presence of hypertension and dyslipidemia was also strongly associated with macrovascular (p=0.005 and p=0.003, respectively) and microvascular (p<0.001) complications. Statin use was significantly more prevalent among those with macrovascular complications (p=0.007). Diabetic foot ulcers were significantly more common in patients with macrovascular (p<0.001) and microvascular (p<0.001) complications compared to their counterparts.

**Table 2:** Determinants of macrovascular and microvascular complications among patients with diabetes

Variable		Macrovascular complications, n (%)		Test value	p-value	Microvascular complications, n (%)		Test value	p-value
		Yes, n=105	No, n=480			Yes, n=251	No, n=334		
Sex	Male	59 (18.1)	267 (81.9)	0.011	0.916	136 (41.7)	190 (58.3)	0.311	0.515
	Female	46 (17.8)	213 (82.2)			115 (44.4)	144 (55.6)		
Nationality	Bahraini	91 (18.2)	408 (81.8)	0.191	0.662	222 (44.5)	277 (55.5)	3.348	0.062
	Non-Bahraini	14 (16.3)	72 (83.7)			29 (33.7)	57 (66.3)		
Smoking	Yes	14 (17.9)	64 (82.1)	0.889	0.641	32 (41)	46 (59)	0.282	0.869
	No	80 (17.4)	379 (82.6)			197 (42.9)	262 (57.1)		
	Ex-smoker	11 (22.9)	37 (77.1)			22 (45.8)	26 (54.2)		
Alcohol	Yes	3 (25)	9 (75)	1.938	0.438	6 (50)	6 (50)	0.833	0.659
	No	102 (17.8)	471 (82.2)			245 (42.8)	328 (57.2)		
Type of diabetes	1	1 (5.3)	18 (94.7)	2.146	0.143	3 (15.8)	16 (84.2)	5.826	0.015
	2	104 (18.4)	462 (81.6)			248 (43.8)	318 (56.2)		
Insulin	Yes	48 (22.7)	163 (77.3)	5.164	0.023	111 (52.6)	100 (47.4)	11.911	<0.001
	No	57 (15.2)	317 (84.8)			140 (37.4)	234 (62.6)		
Metformin	Yes	79 (17.2)	379 (82.8)	0.702	0.402	188 (41)	270 (59)	2.453	0.085
	No	26 (20.5)	101 (79.5)			63 (49.6)	64 (50.4)		
Statins	Yes	94 (20.1)	374 (79.9)	7.254	0.007	207 (44.2)	261 (55.8)	1.572	0.195
	No	11 (9.4)	106 (90.6)			44 (37.6)	73 (62.4)		
Hypertension	Yes	82 (21.1)	306 (78.9)	7.938	0.005	192 (49.5)	196 (50.5)	19.841	<0.001
	No	23(11.7)	174(88.3)			59 (29.9)	138 (70.1)		
Dyslipidemia	Yes	93 (20.4)	362 (79.6)	8.626	0.003	214 (47)	241 (53)	13.915	<0.001
	No	12 (9.2)	118 (90.8)			37 (28.5)	93 (71.5)		
Diabetes control	Yes	36 (15.3)	200 (84.7)	1.950	0.163	90 (38.1)	146 (61.9)	3.420	0.055
	No	69 (19.8)	280 (80.2)			161 (46.1)	188 (53.9)		
Diabetic foot ulcer	Yes	14 (70)	6 (30)	38.096	<0.001	20 (100)	0 (0)	23.115	<0.001
	No	91 (16.1)	474 (83.9)			231 (40.9)	334 (59.1)		

Patients with macrovascular complications were significantly older (p<0.001) and had a longer history of diabetes (p=0.013) compared to those without. Similarly, patients with microvascular complications were older (p< 0.001) and had longer diabetes history (p<0.001). Additionally, laboratory results showed that patients with macrovascular complications had lower total cholesterol (p=0.002), lower low-density lipoprotein (LDL) levels (p=0.009), lower eGFR (p<0.001), and lower hemoglobin levels (p=0.017), whereas patients with microvascular complications, had higher HbA1c (p=0.025), elevated triglycerides (p=0.017), reduced eGFR (p<0.001), and lower hemoglobin (p=0.001; see Table 3).

**Table 3:** Association between participants' age, duration of diabetes, laboratory findings, and cardiovascular complications of diabetes DM - diabetes mellitus; A1c - glycated hemoglobin; LDL - low-density lipoprotein; HDL - high-density lipoprotein; eGFR - estimated glomerular filtration rate

Variable	Macrovascular complications, median±IQR		Test value	p-value	Microvascular complications, median±IQR		Test value	p-value
	Yes, n=105	No, n=480			Yes, n=251	No, n=334		
Age in years	65±10	60±14.5	3.800	<0.001	64±11	59±16	4.499	<0.001
DM duration in years	15±10	10±13	2.310	0.013	15±10	10±10	6.099	<0.001
Fasting blood glucose (mmol/L)	7.3±3.7	7.2±3.2	0.318	0.751	7.4±3.8	7.1±3.0	0.022	0.318
Hemoglobin A1c (mmol/mol)	58±22.75	56±24.00	0.118	0.578	60±24	55±22	1.217	0.025
Total cholesterol (mmol/L)	3.8±1.28	4.1±1.2	2.667	0.002	4±1.3	4±1.2	1.197	0.427
LDL (mmol/L)	2.01±1.08	2.17±0.99	1.695	0.009	2.14±1.01	2.15±1.01	0.738	0.976
HDL (mmol/L)	1.03±0.41	1.09±0.36	1.607	0.101	1.07±0.43	1.09±0.34	0.172	0.417
Triglycerides (mmol/L)	1.5±1.15	1.5±1.00	0.739	0.808	1.7±1.0	1.5±0.9	1.498	0.017
Vitamin B12 (pmol/L)	265.8±153.28	249.3±111.55	1.549	0.138	255.15±130.77	250.35±111.10	1.743	0.404
eGFR (mL/min/1,73m^2)	87±40.75	97±35.00	3.928	<0.001	85±51.5	99±29	6.843	<0.001
Hemoglobin (g/dL)	12.8±2.2	13.3±2.12	2.409	0.017	13±2.3	13.4±2.1	3.509	0.001

In the multivariate logistic regression analysis, higher total cholesterol levels (OR=1.326, p=0.031) and lower eGFR (OR=1.009, p=0.025) were significant predictors of macrovascular complications. In addition, patients with diabetic foot ulcer were approximately 14 times more likely to have macrovascular complications compared to those without foot ulcers (OR=14.286, p<0.001). Patients with type 2 diabetes had 16 times more risk of suffering from microvascular complications compared to those with type 1 diabetes (OR=16.277, p=0.011). Moreover, longer diabetes duration (OR=1.156, p<0.001) and higher triglyceride levels (OR=1.222, p=0.045) were significant risk factors for microvascular diabetic complications. Patients with diabetic foot ulcers had a 27-fold higher risk of developing microvascular complications compared to their counterparts (OR=27.217, p=0.002). Additionally, patients with dyslipidemia had twice the risk of developing diabetic microvascular complications compared to those without dyslipidemia (OR=0.587, p=0.036; see Table 4).

**Table 4:** Final step of the logistic regression analysis of factors associated with macrovascular and microvascular complications among patients with diabetes * Variables entered on step 1: age, diabetes duration in years, insulin, statin use, hypertension, dyslipidemia, total cholesterol (Mmol/L), hemoglobin (G/Dl), diabetic foot ulcer ** Variables entered on step 1: age, type of diabetes, diabetes duration in years, insulin, hypertension, dyslipidemia, hemoglobin A1c, triglyceride level, hemoglobin level, diabetic foot ulcer

Variables		Odds ratio (95% confidence interval)	p-value
Macrovascular complications*	Age (years)	0.978 (0.956-1.002)	0.070
	Statins	0.481 (0.231-1.003)	0.051
	Total cholesterol	1.326 (1.026-1.713)	0.031
	Estimated glomerular filtration rate, eGFR	1.009 (1.008-1.010)	0.025
	Diabetic foot ulcer	14.286 (2.345-34.579)	<0.001
Microvascular complications**	Type of diabetes	16.277 (1.884-140.597)	0.011
	Diabetes duration (years)	1.156 (1.135-1.237)	<0.001
	Hypertension	0.663 (0.427-1.030)	0.068
	Dyslipidemia	0.587 (0.357-0.966)	0.036
	Triglycerides level	1.222 (1.153-1.346)	0.045
	Hemoglobin level	1.123 (0.999-1.263)	0.051
	Diabetic foot ulcer	27.217(3.396-218.159)	0.002

## Discussion

This study aimed to explore the prevalence and predictors of macrovascular and microvascular complications among patients with DM attending primary healthcare centers in Bahrain. The results showed that more than half of the participants had at least one diabetes-related complication. Neuropathy and retinopathy were the most common microvascular complications, while peripheral vascular disease and ischemic heart disease were the most frequent macrovascular complications. The study also found that higher diabetic foot ulcers were associated with macrovascular complications, while type 2 DM, longer disease duration, higher triglycerides, dyslipidemia, and diabetic foot ulcers were significant factors associated with microvascular complications.

The prevalence of diabetes mellitus complications shows considerable variation across published studies. In this study, the prevalence was lower when compared to several regional studies. For instance, some studies from Egypt and Saudi Arabia reported higher rates of both microvascular and macrovascular complications. Specifically, the prevalence of neuropathy in this study was 27% compared to 20% in Egypt and Saudi Arabia, while the prevalence of retinopathy was 15% in the present study compared to 35% and 43% in Egypt and Saudi Arabia, respectively [10,12]. Nonetheless, some studies have reported lower rates of diabetic complications. For example, a study from Saudi Arabia found that only 6% of patients with diabetes had diabetes-related complications, compared to more than half of the patients (51.6%) in the present study who had at least one diabetes-related complication, which represents a significantly higher proportion of patients [11]. The variation in the prevalence of diabetes-related complications could be attributed to differences in population characteristics, settings, and diabetes features. For instance, some studies included newly diagnosed patients, while other studies included all patients. In addition, some studies were done in a community setting, while other studies were done in hospital settings. 

In line with previous studies, this study found that longer diabetes duration was significantly associated with both microvascular and macrovascular complications. Prolonged hyperglycemia results in endothelial dysfunction and oxidative stress, which accelerates vascular injury [14]. Similar associations between diabetes duration and complication risk have been reported in multiple studies across different populations [9,15]. As expected, diabetic foot ulcers were strong predictors of both macrovascular and microvascular complications, reflecting advanced disease and poor peripheral circulation. The pathogenesis of diabetic foot ulcers involves the presence of a combination of neuropathy, poor immunity, and peripheral arterial disease [16].

In the present study, low glomerular filtration rate was a predictor of macrovascular complications. Similar findings were reported in the literature [17]. As glomerular filtration decreases, it indicates widespread endothelial dysfunction, inflammation, and atherosclerosis in the body [18].

Surprisingly, total cholesterol levels were lower among patients with macrovascular complications when compared to their counterparts. This finding could be explained by the use of statins among such patients. The results also showed that dyslipidemia was higher among patients with microvascular complications. The role of high lipid levels in atherogenesis, endothelial dysfunction, and microcirculatory changes was well-established in the literature [19]. Guidelines emphasize intensive management of dyslipidemia among patients with DM for optimal complication prevention [20]. Patients with atherosclerotic cardiovascular complications often require high-intensity statins and lower lipid targets to control their cardiac risks [21].

Although the univariate analysis showed an association between diabetes-related complications and both patients' age and hypertension status, the logistic regression analysis did not reveal any significant relationships for these variables when included in the multivariate analysis. Similar associations, however, have been reported in the literature [8-12].

The present study has several strengths. It included all primary care centers and an adequate sample size. In addition, it assessed all microvascular and macrovascular complications of diabetes and several risk factors. Most outcomes were measured through objective tools like the monofilament test, retina assessment, and laboratory results. However, it also has some limitations. Some variables, such as physical activity, dietary habits, and medication adherence, were not assessed, which may influence complication risk. Although some complications, like stroke, were assessed based on patients' recall and verified from medical records, this might introduce the risk of recall bias, as the medical records included details of the last 10 years only. Moreover, the limited sample size for some variables, like type 1 diabetes and patients with foot ulcers, represents a key limitation of this study, as it may have contributed to inflated odds ratios and reduced the precision of the estimated associations.

## Conclusions

In conclusion, this study revealed that more than half of diabetic patients attending primary healthcare centers in Bahrain had at least one diabetes-related complication, with neuropathy and retinopathy being the commonest microvascular complications, and peripheral vascular disease and ischemic heart disease being the commonest macrovascular complications. Patients with longer diabetes duration, dyslipidemia, and diabetic foot ulcers had higher risks of developing these complications. Although causality cannot be inferred from this cross-sectional study, routine assessment for diabetes-related complications and comprehensive diabetes care, focusing on glycemic and lipid management, renal function, and foot care, remains an essential aspect of primary care practice. The comprehensive care should also include structured patient education and promotion of lifestyle modifications, which are important to empowering patients and improving overall disease management.
